# What could we do differently next time? Australian parents’ experiences of the short-term and long-term impacts of home schooling during the COVID-19 pandemic

**DOI:** 10.1186/s12889-022-12495-4

**Published:** 2022-01-13

**Authors:** Alyssa R. Morse, Michelle Banfield, Philip J. Batterham, Amelia Gulliver, Sonia McCallum, Nicolas Cherbuin, Louise M. Farrer, Alison L. Calear

**Affiliations:** 1 Centre for Mental Health Research, Research School of Population Health, The Australian National University, 63 Eggleston Road, Acton ACT, Canberra, 2601 Australia; 2 Centre for Research on Ageing, Health and Wellbeing, Research School of Population Health, The Australian National University, Canberra, Australia

**Keywords:** Home schooling, Remote learning, Home learning, COVID-19, Pandemic, Parents, Caregivers, Parenting, Family relationships, Social isolation

## Abstract

**Background:**

COVID-19 lockdowns have resulted in school closures worldwide, requiring curriculum to be delivered to children remotely (home schooling). Qualitative evidence is needed to provide important context to the positive and negative impacts of home schooling and inform strategies to support caregivers and children as the pandemic continues. This study aimed to explore the experiences of home schooling caregivers at multiple time-points during the pandemic.

**Methods:**

Data were obtained from a longitudinal survey of a representative Australian sample conducted over 8 waves during 2020 and 2021. Participants who had home schooled at least one child during COVID-19 completed open-ended questions at Wave 4 (May 2020; *n* = 176), Wave 7 (June 2020; *n* = 145), and Wave 8 (March 2021; *n =* 57). Participants were asked to describe what they found positive and challenging about home schooling (Wave 4), what they would do differently if they home schooled their children again (Wave 7), and the longer-term impacts of home schooling on caregivers and children (Wave 8).

**Results:**

91% of participants at Wave 4 reported at least one positive and/or negative aspect of home schooling. At Wave 8, 32% and 29% of participants reported no long-term positive or negative impacts of home schooling respectively. Using a qualitative content analysis approach, six themes were developed from the data, encompassing the impacts of home schooling on parents, and the perceived impacts on children. Impacts on parents included connecting with children, managing the work-life-school balance, and the challenge of home schooling when parents are not teachers. Perceived impacts on children included: quieter and safer learning at home, and the negatives of managing schoolwork load and social isolation. At Wave 7, 56 participants (44%) identified at least one thing they would do differently.

**Conclusions:**

Despite some participants reporting positive experiences associated with home schooling, it remains challenging for many parents and their children. Supports for parents and children engaged in home schooling should provide clear and flexible guidance on how to balance schoolwork with other competing demands, assist parents who lack confidence in supporting their children’s remote learning, and address risks associated with social isolation.

## Background

The COVID-19 pandemic has resulted in school closures worldwide as part of strict physical distancing policies aimed at reducing virus transmission [1]. At their peak in 2020, school closures impacted almost 1.5 billion learners across 151 country-wide closures [2]. In May 2021, 26 country-wide closures continued to impact over 200 million learners [2]. This has sparked concerns for children’s wellbeing, learning, development, and safety [2–5], and required educators to adapt curriculum content to be delivered remotely with support and/or supervision from parents (hereafter termed *home schooling*).

School closures and the move to home schooling has also adversely impacted the parents and caregivers of school aged children. In Australia, parents and caregivers who home schooled their children in the first month of COVID-19 restrictions were found to have significantly higher levels of distress and work and social impairments compared to both parents who were not home schooling and people with no dependent children [6]. Qualitative surveys have found similar challenges, describing increases in stress, anxiety and frustration during periods of home schooling [7, 8]. Parents and caregivers reported experiencing challenges balancing usual work and household duties with increased caring and new teaching responsibilities, and difficulties coping and managing time [7, 8]. For some families, COVID-19 lockdown restrictions and home schooling requirements led to reductions in social connection and instrumental support outside the home, increased strain on family relationships and relationship breakdown within the home [7, 8].

While these challenges inspired increased appreciation for teachers, a lack of school support and school communication were the main factors identified by parents dissatisfied with the home schooling experience [8, 9]. Parents were also concerned about the impact of home schooling on child development, including potential delays in learning progress, increases in daily screen time and deteriorating mental health [7–9].

However, the impacts of home schooling are not universally negative, and current evidence suggests that parents’ feelings about home schooling may often be mixed [7]. Some parents found positives during school closures, including less time spent commuting and more time spent bonding with and getting to know their children, increasing family closeness. The home was also perceived to be a better learning environment for some children [7, 8].

Developing qualitative evidence can provide vital context around the impact of school closures and home schooling, describing parents’ experiences and potential drivers for increased levels of distress. Better understanding the positive and negative aspects of parents’ experiences of home schooling will enable the development of appropriate and effective support mechanisms for parents through ongoing periods of COVID-19 related restrictions and future school closures. Improved support for parental health and wellbeing is likely to positively impact the health and wellbeing of their children. Current qualitative explorations of parent experiences have focused on a single time-point, usually early in the pandemic, using convenience or social media samples [7–9]. The aim of the current study was to build on these findings by exploring experiences and reflections of parents, drawn from a representative sample of Australian adults, at multiple time-points during the pandemic. Data were collected across three time-points: [1] experiences during home schooling, [2] reflections about the home schooling experience when social distancing restrictions were being lifted, and [3] the perceived long term impacts of home schooling approximately 12 months after the first school closures and at the beginning of a new school year.

## Methods

### Participants and procedure

The Australian National COVID-19 Mental Health, Behaviour and Risk Communication (COVID-MHBRC) survey was established to longitudinally assess the impact of the COVID-19 pandemic on a representative sample of Australian adults aged 18 years and over [10]. Participants were required to be able to respond to an online English language survey. The study consisted of eight waves of data collection, administered through Qualtrics Research Services; the first seven waves were completed fortnightly online between March and June 2020, and the eighth was completed in March 2021. Participants were emailed an invitation to complete each survey and could submit it within a one-week window. Participants received up to five reminders to complete a survey during the week of data collection. Quota sampling was used to obtain a sample of the Australian population from market research panels that was representative on the bases of age group, gender and State/Territory. The study was approved by The Australian National University Human Research Ethics Committee (protocol 2020/152), and written informed consent was obtained from all participants prior to participating. The full study protocol is available online (https://psychology.anu.edu.au/files/COVID_MHBRCS_protocol.pdf).

The first wave of data collection commenced on the 28th March 2020 (*N* = 1296). Besides demographics and background variables (collected in Wave 1), data for the current study were drawn from waves four (9–16 May 2020), seven (20–27 June 2020), and eight (2–9 March 2021). Over 73% of the initial sample was retained at Wave 2 (W2; *N* = 969). Attrition across subsequent waves was lower, consistently retaining over 90% wave-on-wave (*N* W3 = 952, W4 = 910, W5 = 874; W6 = 820; W7 = 762), until the 6-month follow-up at Wave 8, where 40% of participants returned to complete the final survey (W8; *N* = 519). Analyses were conducted on a subsample of participants who indicated that they had home schooled one or more children during COVID-19 (*n* W4 = 176, W7 = 145, W8 = 57). Participant characteristics are summarised in Table 1.

**Table 1:** Characteristics of parents/carers who had home schooled one or more children during COVID-19 at Wave 4 (*N* = 176)

	***n***	%
**School level of children**^a^		
Primary school	108	61.4
High school	99	56.3
**Working from home**		
Yes	91	51.7
No	85	48.3
**Gender**		
Male	80	45.5
Female	96	54.5
**Language spoken at home**		
English only	149	84.7
English and another language^b^	25	14.2
Another language only^b^	2	1.1
**Highest completed qualification**		
High school or less	35	19.9
Certificate/Diploma	61	34.7
Undergraduate or higher	80	45.5
**Age (Mean, SD)**^c^	42.8	9.7

^a^Participants could select both responses

^b^Includes 16 languages other than English

^c^Age range: 22–71 years

### Measures

#### Demographics

Participants provided details on their age, gender, language(s) spoken at home, and level of educational attainment at Wave 1. At Wave 4, participants were asked for details about their home schooling experience, including whether their children were in primary school or high school, and whether parents had been working from home.

#### Qualitative questions – home schooling experience

Participants were invited to provide open-ended responses at Wave 4, Wave 7 and Wave 8. At Wave 4, participants were asked to describe what they liked best, or had found most positive, about home schooling, and what they liked least, or had found most challenging, about home schooling. At Wave 7, participants were asked to describe what they would do differently if they needed to home school their children again. Finally, at Wave 8, participants were asked about the longer-term outcomes of home schooling on themselves and/or on their children. They were prompted with three possible examples (learning progress, career progression, family connections), and then asked to describe what they thought the long-term positive and long-term negative outcomes of home schooling had been.

### Analysis

Responses to the qualitative survey items were analysed using a qualitative content analysis approach [11] to identify key themes relating to the positive and negative aspects of home schooling and the lessons learnt from the experience of home schooling. Given the brief nature of participant responses, a manifest analysis was conducted, staying close to the text of the survey responses to focus on describing participants’ experiences as they were reported [11]. Participant responses were inductively open coded by one author (ALC). Subsequently, these codes were developed into themes and subthemes by a second author (ARM). The analysis was regularly discussed with other members of the research team to clarify concepts and finalise themes, and a final review of the thematic structure was conducted by a third author (MB). The research team included parents who had home schooled primary and high school aged children during Australia’s lock-down restrictions, while other team members were parents of non-school aged children or did not have children, providing a range of perspectives.

## Results

At Wave 4, parents were asked to describe what they liked best about home schooling and what they liked the least or found most challenging, and at Wave 8, parents were asked what they thought the long-term positive and negative impacts of home schooling had been on their children and on themselves. At Wave 4, out of 172 respondents, 17 participants (10%) reported that there was nothing positive about home schooling, 16 (9%) reported nothing negative, and 6 participants (4%) fell into both categories. Nine participants (5%) reported that they were not directly involved in their child’s home schooling, due to working outside of the home, not having time, or family separation. Most respondents (91%) provided a brief description of at least one positive and/or negative aspect of home schooling.

Out of 56 respondents at Wave 8, 18 (32%) thought there were no positive long-term impacts, 16 (29%) thought there were no negative impacts, 5 (9%) fell into both categories, and 5 were unsure (9%). One parent elaborated that home schooling had hopefully had no long-term effects because their children had received high quality schoolwork from their school, but time would tell. Another reported that the long-term negative effects were minimal, as their children were quite young. Most parents (82%) provided a short description of at least one negative and/or positive aspect of home schooling. Some specifically commented on the long-term impacts they had observed, others instead reflected on their positive and negative experiences during the home schooling period. These reflections may indicate which benefits and challenges remained important or memorable for parents several months after home schooling had finished.

Six major themes were developed from the data and fell into two categories with three themes in each: the *impacts of home schooling on parents*, and the *perceived impacts of home schooling on children*. Impacts on parents included *connecting with children*, managing the *work-life-school balance,* and the challenge of home schooling when *parents are not teachers*. The perceived impacts on children included: the *quieter, safer learning* of home, and the negatives of *managing schoolwork load* and *social isolation*.

### Positive impacts on parents

#### Connecting with children

Many parents were able to spend more time with their children and reported that this was the best thing about home schooling, both in the short- and long-term. Some parents reported using this extra time to bond, interact, and connect with their children and felt family relationships were closer. Home schooling also allowed parents to see and better understand what their children were learning at school, and how they were learning it. Participants appreciated learning more about their children’s academic progress, strengths, and weaknesses, and some enjoyed watching their children work and seeing how much they could achieve.

*I like that I can keep in touch with their learning and of course spend more time with them.*

*It has been nice to see my daughter undertaking different activities*

Some parents also liked being actively involved in their children’s learning. Parents were able to provide one-on-one support, teach using their own methods, and help their children develop key skills such as literacy. Home schooling was also an opportunity for parents to learn new skills (e.g., using virtual platforms), refresh their own learning, and see education from different perspectives. Some parents also better understood their children’s needs and progress at school.

*Refreshes my memory about what I was taught. I like seeing my kids grasp and understand a concept.*

*Being a part of my son’s learning, especially at an important stage where he’s learning to read and write. Greater appreciation for how smart he actually is.*

In the long term, some parents felt that they now had a closer relationship with their children, and one parent thought that home schooling would be memorable for their children in a positive way: *“I think they will remember the outdoor activities we did routinely together during covid*.”

### Positive impacts on children

#### Quieter, safer learning

The home environment was also reported to have positive impacts on learning. Some parents felt that their home was quieter, more relaxed and had less distractions than the classroom. Additionally, two parents noted that home schooling protected their children from experiencing bullying and peer pressure. Children could complete their work faster or at their own pace, and had less time away from school due to illness. Home schooling also created opportunities for broader learning, including using new technology. In the long term, two parents noted that their children had maintained their learning progress, and another two observed that their children had learned how to be more self-sufficient and structure their workload.

*more time with them, one-on-one teaching; participating in their learning journey; quieter, less distractions; quicker; gives opportunities for broader learning*

Some parents also felt safer keeping their children at home. The main benefit of home schooling for these parents was preventing their children from potential exposure to harm from COVID-19: “*I know they are safe at home*.”

### Challenges for parents

#### Work-life-school balance

Managing and structuring time when balancing work, schooling and home duties was a salient issue for parents. Participants reported struggling to balance work and household duties with the new demands of home schooling. Home schooling was described as time consuming, stressful, and “*so much effort and work*,” and participants reported feeling time poor. It was difficult “*finding enough hours in a day to fit everything in”* and to create and maintain a structured routine. Home schooling was particularly disruptive for parents who also needed to work from home. Supervising and assisting children interrupted parents’ own work and forced one participant to work from home despite needing to be onsite to best fulfil their role. Caring for multiple children attending different schools created additional challenges, as different schools had different requirements and expectations, and children had different needs for care.

*It is stressful when I have to leave my own work. I sometimes take breaks and go for a walk*

*3 different schools, 3 different ages, 3 different ways of doing things and expectations, and still trying to run a household and care for a disabled child*

Home schooling may have also increased appreciation of face-to-face teaching:

*Kids have realised the importance of both daily routines and teachers*

However, there were some differing or mixed experiences of work-life-school balance. Some participants found they had more time in their day and their mornings were more relaxed. Home schooling removed the need to travel to school, prepare school lunches and get children ready for school. One parent felt less tired and another reported their child was able to get more sleep because they did not need to spend time travelling to school.

*Less tiredness. More time in the day, as there is no getting ready or travel time.*

#### Parents are not teachers

Difficulties keeping children focused and engaged with their schoolwork, and “*Getting the kids to actually do the work*” were frequently reported to be the most disliked or challenging aspects of home schooling. Many parents had trouble motivating children and getting them to listen to instruction, and some felt that their child was too dependent on parental support when completing schoolwork. This may have been a particular challenge in the home environment due to distractions competing for children’s attention (e.g., video games, YouTube, playing outside) and work responsibilities competing for parents’ attention.

*… I’m not a teacher and [it] has been hard to motivate kids.*

*When I turn my back he is watching youtube or playing games instead of doing his work.*

Additionally, parents worried about their own ability to understand and teach the content set by schools. Teaching methods have changed since parents attended school and this made it difficult for some parents to help children with their schoolwork. For example, several parents had trouble helping children with maths problems: “*… especially because they do it slightly differently than when I got taught*”. Parents reported difficulty understanding their children’s schoolwork or interpreting what work needed to be completed. Parents also shared concerns about not knowing the answers to their children’s questions, answering questions incorrectly, and not being able to help their children.

*The constant questions... worried that I would give them the wrong answers.*

*Difficulty understanding the school work and not being able to help.*

Given these challenges, home schooling materials that were structured, flexible and had user friendly instructions were appreciated.

Additionally, some parents reported that their home environment was not well set-up for remote learning. Problems with internet access and speed, a lack of knowledge about relevant technology, and a small living space all added difficulty to the home schooling experience.

*Not physically set up in our smaller home for interruption free learning*

Parents were also concerned that children had less opportunities to interact with their teachers. Teachers were seen as less accessible, and less able to answer students’ questions and provide individual-level guidance in the online format. Teachers may also have been struggling with technology, as one parent felt the most challenging thing about home schooling was: “*teachers/school not understanding the technology they’ve been forced to use”*.

While there were many challenges identified, a small number of parents also expressed appreciation for teachers. These parents liked seeing their children’s teachers in action and observed: “*How much the teachers genuinely love the kids*”.

### Negative impacts on children

#### Managing schoolwork load

Many parents felt learning online at home was not the same as learning face-to-face in the classroom. Learning from home was described as not as effective as learning at school, and parents commonly felt that their child would be learning more, or completing more work, in the classroom. Two parents thought there was less variety for children, and two were concerned about the increased amount of screen time that home schooling required. Some parents reported that their children were set too much work by their school or struggled to complete all the set tasks. Others found that their children completed their work quickly and were then left with nothing to do until the next set of tasks arrived.

*We are getting the basics of English, Maths and Science done but even though my daughter is working hard she is not getting to some of the other suggestions or tasks set.* *I know this is fine but I know that she would be getting through more in actual school.*

*While they get their work done, our children are quite bright and so get through their work quickly and are often at a loss of what to do next*

In the long term, some parents noted that their children had fallen behind the expected standards at school or had poorer grades.

*Child has fallen behind the expected standard and has experienced behavioural problems and is still stressed*

#### Social isolation

Parents also observed that their children were missing out on opportunities for direct peer-to-peer interaction, both in educational (e.g., class discussions) and social contexts. The lack of social interaction appeared to be particularly challenging, and children were described as lonely, isolated, stuck at home, and missing their friends. In the long-term, the most commonly reported negative impact of home schooling was the lack of social interaction for children, particularly the isolation of children from their peers, friends and teachers. This topic was raised more frequently at Wave 8 than during the home schooling period (Wave 4: 15/172; Wave 8: 22/56), suggesting that while it was not an issue that parents commonly disliked or found challenging in the moment, they did believe it had an important impact on their children. Long-term, parents noted new behavioural issues, including anti-social behaviour, difficulty focusing, increased stress and difficulty getting children back to school.

*It’s not as effective, there is no collaboration, the lack of social interaction is depressing for my kids*

*Our child misses the interaction with her friends very much.*

### What would parents do differently?

At Wave 7, parents were asked what they would do differently if they needed to home school their children again. Out of 127 respondents, 54 (43%) reported that they would not do anything differently, and 17 (13%) were unsure. Some of these participants elaborated that they felt the home schooling experience went well, was enjoyable, or that there was nothing more they could have done under the circumstances. This suggests that while participants found aspects of home schooling challenging in the moment, many felt there was nothing they would or could have done differently. However, one of the unsure participants specified that they found home schooling quite difficult to manage.

The remaining 56 participants (44%) identified at least one thing they would do differently. Three categories were developed from this data, representing key strategies for improvement: *manage time*, *seek support*, and *be prepared*. Each of these strategies addressed one or more of the challenges described by parents at Wave 4.

#### Manage time

Most participant comments were related to time and time management. Some participants would make more time for home schooling in their day, potentially by starting schoolwork earlier in the day, working from home, asking for different work shifts, or reducing work hours.

*Take time off or reduce hours*

Participants also suggested that they would structure their time better, for example, by organising a routine or following a school timetable. A small number suggested they would be stricter when home schooling. In contrast, there were a small number of participants who indicated that next time they home schooled, they would stress less, be more patient and not try to do everything.

*Make sure they had set tasks to take up the whole day.*

*Not try to do everything*

#### Seek support

Some participants suggested they would seek more external support for home schooling duties, such as hiring a tutor or asking for more help from family members. Parents would also be more proactive when interacting with their children’s school; taking the initiative when contacting teachers, asking for more schoolwork, clarifying the information provided by the school, and providing suggestions for how to improve the remote learning experience. One parent suggested they would seek more social support for their child, making sure they “*have the contact details of more of their school friends so they can communicate socially more.”*

#### Be prepared

A smaller number of comments were related to being more prepared for home schooling in the future. Some of these parents reported that they would acquire updated technology (e.g., a better computer), manage their physical space at home better, or access more online learning resources:

*That's hard to say, it would be nice to just home school and not work, so maybe [would] have used more online resources which were very helpful when I found them*

Others would try to prepare themselves to be more effective teachers by making sure they understood the content of their children’s schoolwork and current learning approaches.

*Do more prep work so I understood what they were learning*

## Discussion

This study provided highly valuable insights into parents’ and carers’ experiences of home schooling their children during the COVID-19 pandemic. While some parents identified no positive or no negative impacts of home schooling, the vast majority (72%) reported some positive and negative consequences on both parents and children. Particular benefits for parents were the ability to spend more time with their children, thus providing greater opportunities to bond and develop closer family relationships, as well as developing a better understanding of their children’s learning. However, parents also reported increased stress associated with the difficulty of managing work-life-school balance, not having enough time to do everything, and juggling the varying needs of multiple children. In contrast, when considering their children’s experience, some parents perceived the calmer, and in some instances psychologically safer, home environment as positive and more suited to a timely and broader learning experience. Although, many parents also felt that their children’s learning at home might have been less effective than learning at school, that their children were given either too much or not enough work to complete, and that they were missing out on important social interactions. Overall, many parents were philosophical about what could be achieved given the unexpected and unforeseeable circumstances, while also providing thoughtful reflections about what could be done differently in the future to better support parents and children.

When initial lockdowns were announced, parents and students received little warning regarding the closure of schools. It was a challenging time requiring rapid adjustment, and yet, strong positive themes emerged from participants on their experiences. Consistent with previous research, many participants reported enjoying increased time with their children and the opportunity to appreciate and understand what they were learning at school [7, 8]. Further, in the current study, some participants reported that building a stronger connection with their child was a long-term benefit from home schooling. Recognising home schooling as an opportunity to strengthen relationships with children is an important and positive way to frame home schooling episodes in the future, and resources have been developed to assist with this [3]. Parents also expressed some unexpected benefits arising from COVID-19, including an increase in available time due to not having to travel or prepare for school; experiences shared by parents and carers in other studies [7, 8]. Helping parents and caregivers to identify and utilise the personal benefits of home schooling may assist them to better manage the challenges and stressors of school closures.

Most challenges parents identified related to the lack of notice or ability to plan for home schooling. Consistent with previous research [7, 8], many parents reported difficulties with scheduling children’s class time and competing demands for time, technological limitations, and lack of support from schools. Addressing some of these challenges may be a key target for interventions aimed at reducing parent and caregiver stress and improving their adjustment to periods of school closure. Nearly half of respondents in the current study indicated that they would do something differently the next time they home schooled, including negotiating to reduce paid work hours and better managing their children’s routines or timetable. Additionally, the need for more support from school staff during closures has been expressed by parents and caregivers in other studies [8, 9]. Our findings indicate that time management and motivational tips, and strategies for managing competing distractions at home could make a valuable contribution to the school resources provided to parents during future home schooling periods. There is also a need for workplaces to recognise the burden created by home schooling requirements, and to provide appropriate parental leave or flexible working arrangements for impacted employees.

Many parents also had difficulties understanding or working with new teaching methods that had changed since they were at school themselves and lacked confidence when answering questions about schoolwork. This may have been particularly challenging or stressful for parents with lower education levels, or for those who speak a language other than English at home, due to lower literacy, numeracy or English language fluency [6, 12]. Recommendations for future periods of home schooling could include teaching materials that are highly structured, flexible, and provide clear instructions for parents. For example, schools could provide a brief video tutorial for parents that outlines the concept or approach being taught that day. More broadly, subsequent periods of home schooling should focus on assessing the capacity and resources of parents to home school, paying attention to socioeconomic disadvantage and access to technology, and not increasing gaps [12, 13].

Several key impacts of home schooling on children were also identified. Positive impacts included the benefits for some children of self-directed learning, away from distractions. However, students with a preference for structured or collaborative learning may have seen less benefit from home schooling, with their preferences potentially mediated by an increased need to use technology for a large proportion of home learning (see, e.g., [14]) . Indeed, increased workloads for children were reported as a negative impact, suggesting that some students may have struggled without the structure and dedicated resources of the classroom environment and collaboration with peers. In contrast, other students completed all available tasks too quickly and were left without enough activities to fill a school day. Providing flexible delivery of curriculum to students, with core and extended activities and opportunities for virtual collaboration may assist with differences in capability for self-directed learning.

Reduced exposure to bullying and negative peer interactions was noted by some parents. Children and adolescents who experience social difficulties or bullying at school may have a greater preference for the home schooling environment. While it is possible that increased technology use may be associated with cyberbullying, other contextual and social factors may have mitigated the risk of bullying [15], and no participants reported increased bullying. While reduced negative peer interactions was seen as a benefit, the reduction in positive social interactions was viewed by many as a major drawback of home schooling. Our long-term data suggest that concerns about social isolation in children and adolescents stemming from the home schooling experience may continue to have impacts for months after students have returned to the classroom. These findings about the mixed social impacts of lockdowns are largely consistent with other research. A recent review reported that the social impacts of school closures on mental health tend to occur after isolation ends and students return to school [4], suggesting that the social disruption of home schooling may have delayed and prolonged effects on adolescent social functioning and peer networks. During school closures in the UK, students with poor mental health and low peer connectedness had greater improvements in mental health [16], indicating that risks and benefits of home schooling are likely to be highly variable depending on context and individual characteristics. Creating physically safe ways for connecting children with teachers and each other may be critical for maintaining social interaction to overcome loneliness. Creative use of technology to support social connection and not just as a tool for learning should be encouraged.

This study had many strengths but also several limitations. The data were collected at the first peak of home schooling in Australia and provide an important baseline against which future home schooling periods can be compared. The individuals surveyed were generally representative of the population and therefore these findings provide rare insights which cannot be obtained by convenience sampling which has been typically used in other studies. However, it is important to recognise that under-privileged groups - those with low income, educational attainment, and employment - were not likely to be adequately represented, although they may have been even more affected by home schooling [13]. Our findings are also limited by being based on data collected over three two-week periods which may not capture the full or lasting experience of parents and carers. Therefore, long-term studies with more frequent longitudinal assessments are needed to determine the extent to which individual experience changes over time and across longer lock-down periods. Finally, the breadth of the qualitative data obtained on home schooling was limited by the nature of the longitudinal cohort project which aimed to assess the impact of the COVID-19 pandemic across several quantitative and qualitative domains. Thus, dedicated in-depth investigations should be conducted to provide more detail on the home schooling experience during a pandemic.

## Conclusions

Despite some participants reporting positive experiences associated with home schooling, it remains a challenging experience for many parents and their children. Supports for parents and children engaged in home schooling should provide clear and flexible guidance on how to balance schoolwork with other competing demands, assist parents who lack confidence in supporting their children’s learning, and address risks associated with social isolation.

## Data Availability

The datasets used and/or analysed during the current study are available from the corresponding author on reasonable request.
